# Kawasaki Disease in a Child With Trisomy 18 Treated With Initial Combination Therapy, Including Cyclosporine

**DOI:** 10.1155/crpe/5048859

**Published:** 2026-02-20

**Authors:** Yasuyuki Sahara, Naomi Yagi, Yoshitaka Watanabe, Koichiro Aoki, Takashi Iwaku, Makiko Tominaga, Hirokazu Ikeda

**Affiliations:** ^1^ Children’s Medical Center, Showa Medical University Northern Yokohama Hospital, Yokohama-shi, Kanagawa, Japan

## Abstract

**Background:**

Kawasaki disease is a systemic inflammatory disorder that is frequently encountered in routine pediatric clinical practice. Conversely, trisomy 18 is a chromosomal disorder with a historically poor prognosis. However, recent advances in medical care have improved its survival, with a reported 1‐year survival rate reaching 29%. Despite this improvement, studies describing the clinical course of individuals with trisomy 18 beyond the first year of life remain scarce. Moreover, to our knowledge, cases of Kawasaki disease with trisomy 18 have not been reported.

**Case Presentation:**

We report a case of a 3‐year‐11‐month‐old girl with trisomy 18 who presented with persistent fever and rash and, subsequently, fulfilled five of the six principal diagnostic criteria for Kawasaki disease. Because intravenous immunoglobulin resistance was predicted based on the Kobayashi score, initial treatment consisted of intravenous immunoglobulin (2 g/kg), aspirin, and oral cyclosporine concurrent with current Japanese guidelines. Defervescence was achieved without complications, and no adverse events were observed during treatment. No coronary artery abnormalities were observed during the acute phase or at 1‐, 3‐, and 6‐month follow‐up evaluations.

**Discussion:**

We present a case of trisomy 18 treated according to Japanese guidelines for Kawasaki disease. Treatment regimen, which included cyclosporine, was safely administered, and the patient experienced a favorable clinical course. To improve the life expectancy of this patient group, it is crucial to accumulate comprehensive natural history, including the efficacy of standard treatments for various pediatric conditions. This will enable timely and appropriate therapeutic interventions.

## 1. Introduction

Kawasaki disease is a systemic inflammatory disorder manifesting as vasculitis, and it is frequently encountered in routine pediatric clinical practice. In Japan, the treatment guidelines for Kawasaki disease have been revised in 2020 [1]. For cases predicted to be resistant to intravenous immunoglobulin, based on scoring systems such as the Kobayashi score [2], combination therapy with cyclosporine or prednisolone is recommended [1].

Alternatively, trisomy 18 (T18) is a chromosomal disorder caused by full or partial chromosome 18 duplication. It is commonly associated with congenital heart disease and other anomalies. Historically, its prognosis was poor, with a median survival of 14.5 days and a 1‐year survival rate of 5.6% [3]. However, recent advances in neonatal intensive care and proactive management of associated congenital heart disease have improved outcomes, with reported 1‐year survival rates reaching 29% [4] and 5‐year survival rates reaching 12.3% [5]. Despite the increase in survival rates for T18, studies describing the clinical course of individuals with T18 beyond the first year of life remain scarce. To our knowledge, no cases of Kawasaki disease with T18 have been reported. We herein report a case of a girl with T18 who developed Kawasaki disease and responded well to initial combination therapy, including cyclosporine.

## 2. Case Presentation

The patient was delivered via elective cesarean section at 37 weeks and 5 days of gestation (birth weight, 1685 g; Apgar scores, 5 and 7 at 1 and 5 min). Prenatal amniocentesis revealed T18, whereas postnatal chromosomal analysis by fluorescence in situ hybridization confirmed full T18. At 9 months, she underwent intracardiac repair for ventricular septal defect. Other congenital anomalies were bilateral grade II vesicoureteral reflux and a right duplicated renal pelvis and ureter.

At 3 years and 11 months, she developed a rash on the trunk and a fever of 38°C, which was accompanied by poor tolerance of enteral nutrition and dehydration, prompting hospitalization on Day 2 of illness. Upon admission, her height was 77.9 cm (−5.5 SD); weight, 7.8 kg (−3.8 SD); body temperature, 38.5°C; heart rate, 128 bpm; respiratory rate, 32 breaths/min; and blood pressure, 114/72 mmHg. She exhibited a healthy facial complexion without conjunctival injection, pharyngeal erythema, lip redness, strawberry tongue, or cervical lymphadenopathy. She had clear breath sounds and regular heart sounds, as well as a soft and flat abdomen. Scattered pinpoint‐sized erythematous macules were found on her face and trunk, but there were no peripheral extremity changes.

Laboratory tests revealed a white blood cell (WBC) count of 8430/μL, C‐reactive protein (CRP) of 1.73 mg/dL, elevated liver enzyme levels (aspartate aminotransferase [AST], 195 IU/L; alanine aminotransferase, 286 IU/L), and normal platelet count, serum albumin, and electrolytes. Furthermore, urinalysis revealed no pyuria. Chest and abdominal radiographs showed no changes compared with previous routine evaluations.

On Day 2, intravenous cefotaxime (150 mg/kg/day) was initiated for suspected bacterial infection. Fever persisted until Day 5, and the patient met five of the six principal criteria for Kawasaki disease, except for lymphadenopathy. Thus, a diagnosis of Kawasaki disease was established. Echocardiography revealed no coronary artery dilatation or related abnormalities. Multiplex polymerase chain reaction (Film Array respiratory and gastrointestinal panels) yielded negative results for all screened infectious agents. Laboratory tests on that day showed a WBC count of 11,450/μL with 81% neutrophils, a platelet count of 199,000/μL, sodium of 138 mEq/L, AST of 45 IU/L (decreased from 195 IU/L on Day 2), and CRP of 6.4 mg/dL. The Kobayashi score was five points (cut‐off ≥ 5), based on the neutrophil count (2 points), platelet count (1 point), and AST level (2 points). The initial treatment included oral aspirin (30 mg/kg/day), intravenous immunoglobulin (2 g/kg/dose), and oral cyclosporine (5 mg/kg/day). Cefotaxime was discontinued. On Day 6, defervescence occurred. On Day 7, the cyclosporine trough level was 51.9 ng/mL. On Day 9, aspirin was reduced to 5 mg/kg/day, and on Day 10, cyclosporine was discontinued. The patient remained afebrile and was discharged on Day 15. Table 1 presents the laboratory data and treatment regimen. During the acute phase, no abnormalities in blood pressure, renal function, or electrolytes were observed. No coronary artery abnormalities were observed during the acute phase or at the 1‐, 3‐, and 6‐month follow‐up evaluations.

**TABLE 1:** Laboratory data and pharmacological treatment from the onset of Kawasaki disease.

Parameter	Units	Day 2 (admission)	Day 3	Day 5 (diagnosis)	Day 6	Day 7	Day 9	Day 13
WBC	/μL	8430	6550	11,450		6680	10,850	9520
Platelet	× 10^4^/μL	20.5	16.4	19.9		19.4	19.1	28.0
Neutrophils	/μL	6320	4770	9360		4110	6990	5600
Albumin	g/dL	4.8	3.6	3.4		2.6	2.7	3.4
Creatinine	mg/dL	0.27	0.22	0.18		0.24	0.21	0.25
Sodium	mEq/L	134	139	138		136	136	136
Potassium	mEq/L	3.9	3.5	2.8		2.9	4.2	4.0
Chloride	mEq/L	100	107	98		99	102	101
AST	U/L	195	61	45		60	37	40
ALT	U/L	286	144	92		69	46	28
CRP	mg/dL	1.73	1.82	6.40		3.11	1.38	0.31
BNP	ng/mL					2210.6	270.1	99.9
Fever		Fever	Fever	Fever	Defervescence			
IVIG	g/kg			2				
Aspirin	mg/kg/day			30	30	30	5	5
Cyclosporine	mg/kg/day			5	5	5	5	

*Note:* AST: aspartate aminotransferase, ALT: alanine aminotransferase, IVIG: intravenous immunoglobulin.

Abbreviations: BNP, brain natriuretic peptide; CRP, C‐reactive protein; WBC, white blood cell.

## 3. Discussion

We herein present a case of a 3‐year‐11‐month‐old girl with T18 who developed Kawasaki disease. An accurate diagnosis of Kawasaki disease requires the exclusion of other conditions. In this case, a comprehensive evaluation excluded alternative diagnoses. After the diagnosis, acute‐phase treatment was promptly initiated according to the Kawasaki disease treatment guidelines [1], which included intravenous immunoglobulin, aspirin, and cyclosporine. This resulted in a favorable clinical response.

The 2020 revision of the Kawasaki disease treatment guidelines recommends the addition of cyclosporine or corticosteroids in the initial therapy for patients predicted to be resistant to intravenous immunoglobulin [1]. This recommendation is based, in part, on evidence from major randomized controlled trials [6, 7]. The RAISE study demonstrated the efficacy of intravenous immunoglobulin plus prednisolone in patients at high risk of intravenous immunoglobulin resistance; however, children with chromosomal anomalies were explicitly excluded from that study [6]. Conversely, the KAICA trial evaluated the combined use of intravenous immunoglobulin and cyclosporine in similar high‐risk patients, and chromosomal disorders were not listed among its exclusion criteria [7]. Although children with T18 were not specifically included, they were not categorically excluded in the KAICA trial. Because the patient was predicted to be resistant to intravenous immunoglobulin based on the Kobayashi score, we initiated combination therapy including cyclosporine, rather than standard intravenous immunoglobulin alone, in accordance with Japanese guidelines. In the present case, cyclosporine was administered as part of the initial treatment according to the guidelines, and acute‐phase symptoms improved without adverse effects. No coronary artery abnormalities were observed during the acute phase and at the 1‐, 3‐, and 6‐month follow‐up evaluations.

We describe a case of T18 treated according to Japanese guidelines for Kawasaki disease. The treatment regimen, which included cyclosporine, was safely administered, and the patient experienced a favorable clinical course. As this represents a single successful instance, further evidence is required to determine the generalizability of this approach to other patients with T18 who present with multiple comorbidities. To improve the life expectancy of children with T18, it is necessary to accumulate a comprehensive natural history—including the efficacy of standard treatments for various pediatric conditions—to facilitate timely and appropriate therapeutic interventions.

## Author Contributions

Yasuyuki Sahara, Naomi Yagi and Yoshitaka Watanabe contributed to the conception, drafting, and critical revision of the manuscript.

Koichiro Aoki, Takashi Iwaku and Makiko Tominaga contributed to the conception and critical revision of the manuscript.

Hirokazu Ikeda supervised the conception, drafted the manuscript, and critically revised the manuscript.

## Funding

No funding was received for this manuscript.

## Disclosure

All authors have thoroughly reviewed and approved the final manuscript and acknowledge accountability for all aspects of the work.

## Ethics Statement

Approval of this study was granted by the ethics committee of the Showa Medical University School of Medicine (CR2025019).

## Consent

The participant’s parent provided written approval for publication.

## Conflicts of Interest

The authors declare no conflicts of interest.

## Data Availability

The data that support the findings of this study are available from the corresponding author upon reasonable request.
